# Mental Health and Personality Traits during COVID-19 in China: A Latent Profile Analysis

**DOI:** 10.3390/ijerph18168693

**Published:** 2021-08-17

**Authors:** Mei Li, Md Zahir Ahmed, Fatema Akhter Hiramoni, Aibao Zhou, Oli Ahmed, Mark D. Griffiths

**Affiliations:** 1 School of Psychology, Northwest Normal University, Lanzhou 730070, China; psylimei@gmail.com (M.L.); ahmedzahirdu@gmail.com (M.Z.A.); 2 School of Education, Lanzhou City University, Lanzhou 730070, China; 3 Department of Economics, Sheikh Hasina University, Netrokona 2400, Bangladesh; fatema.akther@shu.edu.bd; 4 Department of Psychology, University of Chittagong, Chattogram 4331, Bangladesh; oliahmed_polash131@cu.ac.bd; 5 Psychology Department, Nottingham Trent University, Nottingham NG1 4FQ, UK; mark.griffiths@ntu.ac.uk

**Keywords:** COVID-19, personality, latent profile analysis, mental health, China

## Abstract

During the COVID-19 pandemic, mental health problems have increased and are likely to be influenced by personality traits. The present study investigated the association between personality traits and mental health problems (anxiety, depression, post-traumatic stress syndrome (PTSD) symptoms, and obsessive–compulsive disorder (OCD) symptoms) through the person-centered approach because this has some advantages over the variable-centered approach. The data were collected from a sample of 765 Chinese citizens who participated in an online survey in October 2020. Latent profile analysis identified three latent personality profiles—highly adaptive, adaptive, and maladaptive. Highly adaptive individuals had higher extroversion, agreeableness, conscientiousness, openness, and lower neuroticism, while maladaptive individuals had lower extroversion, agreeableness, conscientiousness, openness, and higher neuroticism. Multivariate analysis of variance results showed that individuals with highly adaptive profiles had lower anxiety, depression, and PTSD symptoms compared to individuals with adaptive and maladaptive profiles. The findings of the present study indicate mental health professionals would benefit from formulated intervention plans given the association between latent personality profiles and mental health problems.

## 1. Introduction

The novel coronavirus disease 2019 (COVID-19) was first diagnosed in Wuhan, China, in late December 2019 [1]. Although the virus emanated from China, the outbreak was controlled very effectively by the Chinese authorities. In February 2020, cases of new infections in China started decreasing rapidly and the last death from COVID-19 was reported on 17 May 2020 [2], and no further waves of infection have been reported. After controlling the new infection and detruncating down the transmission chain, the Chinese government has focused on national resumption and rehabilitation programs in the post-COVID-19 pandemic period.

The aftermath of COVID-19 pandemic has been described as the “tsunami” of mental illness [3]. In mid-April 2020, 4.5 billion or 58% of the world’s total population had experienced partial or full confinement in the forms of lockdown and social distancing together [4]. Arguably, the financial and mental reverberations induced by COVID-19 measures (e.g., lockdowns, home quarantining, business/education closures, etc.) are extensive and significantly considered as the biggest psychological experiment ever conducted on earth [5].

Consequences of any global public health emergency, such as the COVID-19 pandemic, can affect the mental health well-being of individuals for months or years to come [6]. Mental health could be vulnerable during both the pandemic and post-pandemic period among both the individuals with pre-existing psychological problems as well as those with no previous history of psychological disorders [7]. Individuals affected may develop depression, sleep disorders, or post-traumatic stress disorder (PTSD) immediately after experiencing the pandemic. Such mental health problems can be sustained from months to several years [8,9]. Pandemic-induced quarantine and physical distancing could lead to social withdrawal symptoms including avoidance of crowded places, over-cautious behavior, frequent hand washing, etc. In addition, immobility could also lead to long-term psychological symptoms including depression, anxiety, PTSD, alcohol abuse, irritability, etc. [10]. Moreover, for a minority of individuals, the pandemic may increase thoughts of suicidal ideation, suicide attempts, and suicide. Quarantine, fear of being infected, and economic loss are all factors that can increase psychological distress and may increase the risk of suicidal behavior. A recent systematic review reviewed studies that reported suicidal behaviors during the outbreak of different infectious diseases, including severe acute respiratory syndrome, Ebola virus disease, etc. [11]. The authors reported an association between the outbreaks of infectious diseases and increased suicide attempts. Ammerman et al. [12] suggested an association between COVID-19 related experiences and past-month suicidal ideation and suicide attempts. They also reported that *“a significant proportion of those with recent suicidal ideation explicitly link their suicidal thoughts to COVID-19”* (p. 32).

It is evident that the patterns in individual differences (e.g., feelings, thought processes, and approaches towards the pandemic) are likely to predict how individuals respond to the COVID-19 pandemic [13]. Considering the unprecedented circumstances of the COVID-19 pandemic and the unprecedented preventive measures that have been taken to inhibit and control its infection, individuals with specific personality traits might be responsive to such public health emergencies in different ways [14]. The perception of external stressors may depend to some extent on personality traits, which ultimately predict how mental health might be affected during the crisis [15]. Since personality traits contribute to an individual’s cognitive, emotional, and behavioral differences, it could determine the pandemic reaction [16]. Previous studies have shown an association between personality traits and the onset of mental health conditions [17,18,19].

In relation to the Big Five personality traits, neuroticism and extraversion are the traits that have been most consistently associated with mental health well-being and loneliness during the pandemic [20,21]. Neurotic individuals are prone to negative emotional reactions and distress during periods of uncertainty [22]. Neurotic individuals have also been found to exhibit a significantly greater level of mental illness due to the COVID-19 induced loneliness compared to individuals scoring high on extraversion, agreeableness, openness, and conscientiousness [20]. Neurotic individuals are also more likely to engage in risky behaviors (e.g., cigarette smoking, alcohol drinking, having unprotected sex, engaging in delinquent activities, etc.) to get relief from stressors [23], and consistent vigilance induced by the anxiety leads to adopting strict precautionary behaviors [24].

On the other hand, extraversion is characterized by social connectedness [25] and participating in social activities that enhance their social network [26]. Therefore, extroverts receive more social support during any crisis event [27]. Extroverts sustain positive mental effects for longer periods than introverts, especially while experiencing an emergency [28]. An open individual is open-minded, less fearful, and curious about new things, which leads to less depression and anxiety. Conscientiousness is highly correlated with compliance to health advice and civic responsibilities, which helps to minimize infection risk. Moreover, an individual with high agreeableness is usually warm and friendly which helps them to get more social support from others during a crisis [29].

Previous studies have shown that higher openness and lower neuroticism are significantly associated with behaviors such as social distancing and taking precautionary measures [30]. During the pandemic, individuals with low extraversion and high conscientiousness tend to avoid social gatherings and perceive sanitizing as essential [31]. Blagov [32] found that individuals with low extraversion and higher neuroticism, higher agreeableness, and higher conscientiousness tend to adhere to physical distancing and precautionary behaviors (e.g., mask wearing, frequent hand washing). Bogg and Milad [33] suggested low neuroticism, higher extraversion, higher agreeableness, higher openness, and higher conscientiousness were significantly associated with precautionary behaviors among individuals.

### 1.1. Person-Centered Approaches

Personality research often focuses on the isolated personality traits and their association with other factors utilizing a “variable-centered” approach [34]. The whole population is being assumed to be homogenous in a variable-centered approach. This approach averages the association between personality traits and other variables to the whole population [35]. However, this approach overlooks mutual relationships among personality traits. In contrast to a variable-centered approach, a person-centered approach assumes population heterogeneity and identifies homogenous subgroups within the population. This approach is able to provide greater insight about the underlying mechanism that produces both within-person variation and between-person differences across the observed dimensions [36]. A person-centered approach describes how personality traits are distributed in these identified homogenous subgroups.

It is debatable as to what constitutes the best approach in relation to personality types. Personality types utilizing a person-centered approach were first described by Robins et al. [37] in the mid-1990s and were named ego resilients, overcontollers, and undercontrollers. Over the following decades, a number of studies explored these personality types among different samples, including children and adults in general populations and clinical populations [38,39,40,41,42]. These studies reported mixed results with some studies replicating these personality types, some not, and some reporting more than three types. These three personality types have not been replicated in studies utilizing the HEXACO model of personality [43,44,45]. As far as the present authors are aware, only three studies have assessed personality types using HEXACO traits. Ashton and Lee [43] reported that there was *“no clear clustering of individuals within the space of the six HEXACO-PI dimensions”* (p. 185). Islar et al. [45] identified four types (resilient, overcontrol, undercontrol, and brittle) and Daljeet el al. [44] identified five personality types (socially considerate, self-confident, goal-oriented, withdrawn, and maladjusted). In addition to replicability, incremental validity is another area of debate. Some researchers have investigated the incremental validity of personality types over traits and have also reported mixed findings. For example, Costa et al. [46] and Asendorpf [47] failed to find evidence of higher incremental validity of personality types over traits. On the other hand, Asendorpf and Denissen [48] and Hart et al. [49] found incremental validity of types over traits with longitudinal data.

Although there are debates regarding personality types that have been explored utilizing a person-centered approach, these have some obvious advantages. For example, categorization based on personality types is the organization of individuals at a high level of abstraction. This approach shifts attention to the ways in how personality traits are organized within individuals. While humans interact with environmental stimuli, they interact as a whole rather than a single trait. Moreover, a personality type approach works as a moderator variable in the association between the perception of COVID-19 pandemic and mental health problems [41]. Therefore, the present study utilized a person-centered approach to identify personality types.

Several studies have used the person-centered approach to predict criminality [50] and prejudice [51]. In a recent study, Ahmed et al. [52] utilized this approach to identify individuals who had higher COVID-19 fear, perceived stress, and poor sleep quality. They found that individuals with a maladaptive personality profile (high neuroticism, low extraversion, low agreeableness, low conscientiousness, and low openness) had higher COVID-19 fear, perceived stress, and poor sleep quality compared to those having adaptive (moderate levels of neuroticism, extraversion, agreeableness, conscientiousness, and openness) and highly adaptive (low neuroticism, high extraversion, high agreeableness, high conscientiousness, and high openness) personality profiles.

### 1.2. The Present Study

Based on the aforementioned discussion concerning the association between personality traits and mental health problems, it is assumed that the likelihood of developing and increasing mental health problems would be associated with different personality traits during the COVID-19 pandemic period. Literature has suggested specific personality traits have an important role in the development of most anxiety disorders [53]. Anxiety disorders are often multifactorial, mostly associated with specific personality traits [54]. It is also evident that some personality traits are susceptible to depression and other depressive disorders [55]. The severity of PTSD symptoms has also been found to be positively correlated with neuroticism, Type D (distressed) personality trait, novelty-seeking, and harm avoidance [56]. Traumatized individuals need specific intervention plans that take personality dysfunction into account [57]. In relation to the COVID-19 pandemic, the necessity of personal hygiene has been highlighted [58]. However, for a minority of individuals, personal hygiene disproportionately affects them and has led to the development of OCD symptoms during the COVID-19 pandemic [59]. As vulnerability indicators, different personality traits have been associated with OCD symptoms in previous studies [60,61]. To the best of the authors’ knowledge, no previous study has assessed the association between personality traits and mental health problems (anxiety, depression, mental well-being, PTSD symptoms, and OCD symptoms) during the pandemic utilizing a person-centered approach.

Therefore, the present study assessed the association between personality traits and mental health of individuals during the pandemic utilizing latent profile analysis (LPA) because person-centered approaches have relative advantages over variable-centered approaches. The main objective of the present study was to assess the association between personality traits and mental health problems among Chinese individuals during the COVID-19 pandemic, and latent profile analysis was carried out to explore Chinese individuals’ latent personality profiles. The association between identified profiles and mental health problems was then examined.

## 2. Methods

### 2.1. Participants

In this present study, data were collected through an online survey utilizing *Tencent.* The *Tencent* survey link was sent to the participants via *WeChat* and *QQ,* the most popular two social media platforms of China. The online survey was conducted between 17 October 2020 to 26 October 2020, where a total of 765 participants completed the survey. Respondents of the online survey received a digital gift voucher worth 10 Chinese Yuan. The participants’ ages ranged from 18 years and 69 years (M = 31.97, *SD* = 10.43 years). Participants’ socio-economic characteristics are presented in Table 1.

### 2.2. Measures

Participants in the present study completed an online survey comprising the Generalized Anxiety Disorder Assessment (GAD-7: Original version [62]; Chinese version [63]), Patient Health Questionnaire (PHQ-9: Original version [64]; Chinese version [63]), PTSD Checklist—Civilian Version (PCL-C: Original version [65]; Chinese version [66]), Yale-Brown Obsessive Compulsive Scale (Y–BOCS: Original version [67]; Chinese version [68]), and Big Five Inventory-10 (BFI-10: Original version [69]; Chinese version [70]). Additionally, a separate section asking questions concerning socio-demographic information (age, gender, marital status, residence, education level, profession, and monthly income) were included in the online survey.

*Generalized Anxiety Disorder Assessment (GAD-7)*: The GAD-7 is a seven-item screening tool that assesses severity of generalized anxiety disorder. Participants are asked to rate their severity of anxiety symptoms over the past two weeks (e.g., *“Feeling afraid as if something awful might happen”*). Items are responded to on a four-point scale from 0 (*not at all*) to 3 (*nearly every day*) with scores ranging from 0–21, where scores of 5, 10, and 15 denote mild, moderate, and severe anxiety, respectively. In the present study, the scale had very good internal consistency reliability (*ω* = 0.884, *α* = 0.883).

*Patient Health Questionnaire (PHQ-9)*: The PHQ-9 is a nine-item screening tool developed to assess depression severity. Participants were asked to rate how often they were bothered by several problems over the past two weeks (e.g., “Little interest or pleasure in doing things”). Items were responded to in a four-point scale from 0 (*not at all*) to 3 (*nearly every day*) with scores ranging from 0–27, where scores of 5, 10, 15, and 20 denote mild, moderate, moderately severe, and severe depression, respectively. In the present study, the scale had very good internal consistency reliability (*ω* = 0.868, *α* = 0.867).

*PTSD Checklist—Civilian Version (PCL-C)*: The PCL-C is a 17-item screening tool used to assess traumatic experience among the general population over the past month (e.g., *“Repeated, disturbing memories, thoughts, or images of a stressful experience from the past”*). Items are responded to in a five-point scale from 1 (*not at all*) to 5 (*extremely*) with scores ranging from 17–85, where scores ranging from 17–29 denote ‘little or no severity’, 28–29 denote ‘some PTSD symptoms’, 30–44 denote ‘moderate to moderately high severity of PTSD symptoms’, and 45–85 denote ‘high severity of PTSD symptoms’. In the present study, the scale had excellent internal consistency reliability (*ω* = 0.930, α = 0.929).

*Yale–Brown Obsessive Compulsive Scale (Y–BOCS)*: The Y–BOCS is a 10-item screening tool used to assess the severity of the symptoms of obsessive–compulsive disorder (e.g., *“How much distress do your distress do your obsessive thoughts cause you”?*). Items are responded to in a five-point scale from 0 (*no symptoms*) to 4 (*extreme symptoms*), with scores ranging from 0 to 40. Higher scores suggest greater severity of obsession–compulsion symptoms. In the present study, the scale had very good internal consistency reliability (*ω* = 0.858, α = 0.857).

*The Big Five Inventory-10 (BFI-10)*: The BFI-10 is a 10-item scale assessing the Big Five personality traits (two items for each personality trait). Participants are asked to rate how each statement describes their personality (e.g., *“I see myself as someone who is reserved”*). Items are responded to in a five-point scale from 1 (*strongly disagree*) to 5 (*strongly agree*). Total scores for each trait range between 2 and 10. In the present study, inter-item correlation of each scale ranged between 0.367 and 0.537 (recommended range between 0.15 and 0.50 [70]).

### 2.3. Statistical Analysis

Latent profile analysis (LPA) was carried out for the Big Five personality traits. Two-class solution to four-class solution models were run in LPA and each model was compared to one-class less solution to identify the best fit model. The best fitting model was identified using the following model fits: Akaike’s Information Criterion (AIC), Bayesian Information Criterion (BIC), Sample-Adjusted BIC (SABIC), entropy, Lo-Mendell-Rubin adjusted likelihood ratio test (LMRT), and average probability of class membership. Lower AIC, BIC, and SABIC values are indicative of better fitting models. An entropy value of 0.80 suggests a better fitting model, although there is no clear cut-off value for entropy. Regarding LMRT, a non-significant LMRT value of model suggests that a one-class lower model is the better fitting model. Regarding average probability of class membership, the average posterior class membership probability of ≥0.7 is acceptable [71]). Finally, multivariate ANOVA (MANOVA) was carried out to assess the association between explored latent personality profiles and mental health problems.

## 3. Results

The fit statistics (AIC, BIC, SABIC, entropy, Lo-Mendell-Rubin adjusted likelihood ratio test (LMR LRT), class size, etc.) of the two-class, three-class, and four-class solutions latent profiles are presented in Table 2. Among solutions, the four-class solutions had lowest AIC (14.358.69), BIC (14,488.60), and SABIC (14,399.69) values compared to three-class solution (14,445.30, 14,547.38, 14,477.52, respectively) and the two-class solution (14,784.20, 14,858.43, 14,807.63, respectively). However, these fit statistics were not conclusive. The entropy value of the four-class solution (0.77) was lower than the recommended value (0.80). The two-class solution and three-class solution had satisfactory entropy value (>0.80). The next considered fit statistics were the LMRT values of each solution. Significant LMRT value of two-class solution (*p* < 0.001) suggested that the two-class solution fitted better than the one-class solution.

Similarly, the significant LMRT value of the three-class solution (*p* = 0.001) lent support to the three-class solution over the two-class solution. Moreover, the non-significant LMRT value of four-class solution also lent support to the three-class solution. Moreover, class sizes of the three-class solution were satisfactory, ranging between 23% (latent profile one) and 50.5% (latent profile two). The average class probabilities of the three-class solution were also satisfactory, ranging from 0.895 (latent profile one) to 0.923 (latent profile three). Overall, the fit statistics suggested three latent personality profiles that underlay the present study’s data.

Due to controversy concerning the validity of personality dimensions vs. types, incremental validities of both were assessed and are presented in Table 3. In Model 1, we first entered profiles (dummy coded) as predictors and mental health variables as outcomes and found R^2^ ranged between 0.006 (for OCD symptoms) to 0.034 (for PTSD symptoms). Then, Big Five personality traits were entered into the model and found significant ΔR^2^ in depression (*p* < 0.05) only. In Model 2, we entered traits first and found R^2^ ranged between 0.017 to 0.043. Then, profiles were entered into the model and found significant ΔR^2^ in anxiety (*p* < 0.05) only. These results suggested that types did not have incremental validity over dimension and vice versa (i.e., dimensions did not have incremental validity over types).

**Figure 1:**
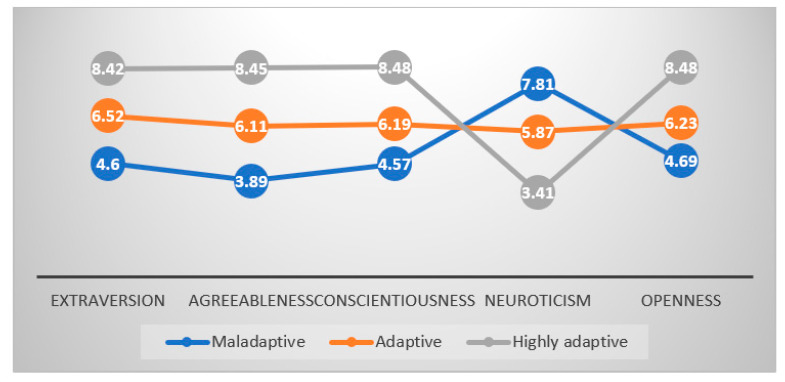
Mean comparisons of the three identified latent profiles.

Table 4 shows the descriptive statistics (means and standard deviations) of the three latent profiles. Individuals in the first profile had lower extraversion, lower agreeableness, lower conscientiousness, lower openness, and higher neuroticism compared to other two profiles. Individuals in the second profile had moderate levels of each of the Big Five personality traits. Individuals in the third profile had higher extraversion, higher agreeableness, higher conscientiousness, higher openness, and lower neuroticism. Based on the existing literature [39,52], the first profile can be labeled as ‘maladaptive’, the second profile as ‘adaptive’, and the third profile as ‘highly adaptive’. Figure 1 shows a comparison among latent profiles in personality traits.

### Comparison among Latent Profiles

Before performing MANOVA, its assumptions (multivariate normality and homogeneity of covariance matrices) were examined. Multivariate normality was assessed using the standard multivariate kurtosis (std M-K). Std M-K with a value less than 5 conforms to the multivariate normality [72]. The std M-K value was 3.57, which was lower than the recommended value. Homogeneity of covariance matrices was assessed using Box’s test (Box’s M = 120.66, F = 1.25, *p* = 0.057). Std M-K value and Box’s test values suggested the suitability of MANOVA for these data. Differences in mental health status (anxiety, depression, PTSD symptoms, and OCD symptoms) among three latent profiles are presented in Table 5. Table 5 also shows mean and standard deviation of post-COVID-19 outbreak anxiety, depression, PTSD symptoms, and OCD symptoms scores of three latent profiles. MANOVA results (Pillai’s *F* (12, 1516) = 7.078, *p* < 0.001) presented in Table 5 show significant mean differences among the three latent profiles in relation to symptoms of anxiety (*F*_(2, 762)_ = 10.15, *p <* 0.001, partial eta squared = 0.028), depression (*F*_(2, 762)_ = 5.04, *p* = 0.003, partial eta squared = 0.015), and PTSD (*F*_(2, 762)_ = 13.22, *p* < 0.001, partial eta squared = 0.034).

Post hoc results (Table 6) suggested that individuals with maladaptive personality profiles had a significantly higher mean difference in anxiety (mean difference = 2.05, *p* < 0.001, 95% CI (0.94, 3.16)), depression (mean difference = 0.23, *p* = 0.003, 95% CI (0.65, 3.20)), and PTSD symptoms (mean difference = 6.68, *p* < 0.001, 95% CI (3.72, 9.64)) compared to individuals with a highly adaptive profile. Table 5 also shows that individuals with adaptive personality profile had a significantly higher mean difference in anxiety (mean difference = 2.12, *p* < 0.001, 95% CI (1.19, 3.06)), depression (mean difference = 1.70, *p* = 0.002, 95% CI (0.62, 2.77)), and PTSD symptoms (mean differences = 5.87, *p* < 0.001, 95% CI (3.37, 8.36)) compared to individuals with a highly adaptive profile.

## 4. Discussion

Personality is an important factor in how individuals deal with their environment, even in emergencies. The present study investigated the association between personality traits and mental health problems among Chinese individuals during the COVID-19 pandemic period, utilizing latent profile analysis. Person-centered approaches (i.e., latent profile analysis) had some obvious advantages over variable-centered approaches. Therefore, a person-centered approach was utilized to assess the association between personality and mental health status concerning COVID-19.

Latent profile analysis identified three latent personality profiles—highly adaptive (lower neuroticism and higher scores in the other four traits), adaptive (moderate scores in all traits), and maladaptive (higher neuroticism and lower scores in the other four traits). Results in the present study regarding incremental validity showed that no traits or types were superior to one another. However, previous studies have shown that personality dimensions have higher incremental validity than types in cross-sectional studies and that personality types have higher incremental validity than dimensions in longitudinal studies [46,47,48,49]. Ahmed et al. [52] also reported similar personality profiles utilizing similar personality assessment tools. Traits’ scores for each profile are also almost similar to profiles suggested by Fisher and Robie [73]. Therefore, these similar profiles’ labeling was utilized in the present study (maladaptive, adaptive, and highly adaptive). However, this personality classification is inconsistent with a number of previous studies [38,74,75]. Inconsistency among studies regarding profile classification might be due to different personality assessment tools utilized in different studies. Quantitative differences among latent profiles in Big Five traits lend support to the newly developing theory of the general factor of personality (GFP: (76)). Considering the intensity of the GFP, highly adaptive individuals are higher in this factor, adaptive are moderate in this factor, and maladaptive are lower in this factor.

MANOVA results showed that individuals having highly adaptive profiles had lower anxiety, depression, PTSD, and OCD symptoms in comparison to individuals with over-controlled and under-controlled personality profiles during this COVID-19 pandemic. These differences in mental health status among personality profiles suggest that there is a strong association between the general personality factor and individual psychological responses to a stressful situation. Individuals with higher GFP had lower anxiety, depression, PTSD, and OCD symptoms compared to individuals with moderate or lower GFP. Previous studies have suggested that higher GFP is associated with high life satisfaction, happiness, good quality of life, good mental health in relation to work, and high job self-efficacy [73,76,77]. Overall, the mental health of people having highly adaptive personality profiles is relatively in good condition compared to people who have the other two personality profiles. Overall personality is an important underlying factor that plays a role in who was affected more and who was not, in terms of mental health. People who have a highly adaptive profile would be less likely to develop symptoms of mental health problems in the post-COVID-era.

The clinical diagnosis of depression, anxiety, or other affective disorders is probably highly pre-disposed by personality features [78]. Often, neurotic individuals are prone to irrational fear that accelerates psychological distress. Neuroticism predicts poor mental health, resulting in lower resilience [79]. Since neuroticism leads to negative emotions and thoughts, it predicts internalizing problems such as anxiety and depression [80]. Neuroticism increases mental health problems bi-directionally because it facilitates internalizing issues and creates emotional problems [81]. Neuroticism and introversion tend to increase the risk of mental health problems and lower psychological well-being [82]. Extraversion, agreeableness, conscientiousness, and openness help enhance the resilience in coping with the COVID-19 pandemic [83]. Given these associations, it is not surprising that the present study found that over-controlling individuals experienced a higher level of distress symptoms than under-controlling individuals and highly adaptive individuals in the present study.

## 5. Limitations and Recommendations

The present study has several limitations. First, personality traits were assessed utilizing a ten-item scale (two items for each subscale). A short personality scale is incapable of covering broad aspects of each underlying construct. Therefore, it is impossible to assess most of the aspects of each trait utilizing only two items. Second, the research data for the present study were collected via a cross-sectional online survey, which may lead to social desirability and memory biases among the participants as well as problems in trying to determine any causality between any of the variables studied. Third, the study examined the association between the personality traits and mental health issues among Chinese individuals during the COVID-19 pandemic phase. However, the present study lacks evidence in ascertaining when mental health problems first began or the extent to which the mental health problems were as a direct result and/or exacerbated by the COVID-19 pandemic. Fourth, the present study did not assess whether participants were affected by COVID-19 and/or by “stay-at-home” public directives. The present study provides an associational link between mental health problems and the pandemic. Because of these limitations, readers of the present study should be cautious when trying to generalize the findings. Moreover, further longitudinal studies, including longer and more comprehensive scales for assessing ‘Big Five’ personality traits, should be carried out to better understand the associations found in the present study.

## 6. Implications

The association between personality traits, mental health well-being, and health behavior are evident in the existing literature [84,85]. Mental health research often addresses personality traits as the key component in the development of such mental health problems [86]. Without considering the personality traits and other individual differences, it is difficult to formulate a better intervention plan for mental health professionals. The findings of the present study will help in developing better interventions. During turbulent crises, such as the COVID-19 pandemic, mental health is impacted [87]. Apart from the physical health issues, mental health has been negatively impacted due to social isolation, quarantines, lockdown, and restrictions on movement during the COVID-19 pandemic [88]. Literature suggests that some individuals are prone to excessive mental health distress in the wake of pandemics, which significantly and negatively affects their mental health well-being [89]. Regular life events have been restricted through a ‘new normal’, so it is extremely important to study how latent personality traits influence mental health distress during and after the COVID-19 pandemic.

## 7. Conclusions

To date, there is little literature examining the person-centered approach in studying personality traits and their association with psychological distress during the COVID-19 pandemic period. The present study demonstrated an association between personality traits and mental health distress (anxiety, depression, PTSD symptoms, and OCD symptoms) during the COVID-19 pandemic in China, utilizing a person-centered approach. By utilizing the person-centered approach, the homogeneity of personality subtypes and their correlations with psychological distress were identified. By profiling personality latently, the study found that ‘highly adaptive’ individuals had higher extraversion, higher agreeableness, higher conscientiousness, higher openness, and lower neuroticism. These individuals reported fewer mental health problems than ‘over-controlled’ and ‘under-controlled’ individuals. Mental health professionals would be benefit from the findings of this present study. These findings would help them to formulate intervention plans emphasizing the association between latent personality profiles and mental health problems.

## Figures and Tables

**Table 1:** Socio-demographic characteristics of the participants.

Variables	Groups	Frequency (%)
**Gender**	Female	603 (52.6%)
	Male	543 (47.4%)
**Education**	Junior school and below	135 (11.8%)
	High school/technical secondary school/technical school	355 (31%)
	University degree (specialized)	379 (33.1%)
	Bachelor	260 (22.7%)
	Masters	17 (1.5%)
**Marital Status**	Unmarried	174 (15.2%)
	Married	937 (81.8%)
	Divorced	30 (2.6%)
	Widow/Widower	5 (0.4%)
**Profession**	Student	83 (7.2%)
	Full time employee	391 (34.1%)
	Part time employee	243 (21.2%)
	Business management	104 (9.1%)
	Self employed	203 (17.7%)
	Unemployed	103 (9.0%)
	Other	19 (1.7%)
**Monthly Income**	0–5000 Yuan	917 (80.1%)
	5001–10,000 Yuan	152 (13.2%)
	10,001 and above Yuan	49 (4.3%)
	Don’t want to disclose	28 (2.4%)

**Table 2:** Model fit indices, class size, and average class probabilities for most likely latent class membership by latent class.

Solutions	AIC	BIC	SABIC	Entropy	LMRT (*p* Value)	Class Size	Average Class Probabilities for Most Likely Latent Class Membership by Latent Class			
							1	2	3	4
2	14,784.20	14,858.43	14,807.63	0.84	1234.19 (<0.001)	484 (63.3%)	0.956	0.044		
						281 (36.7%)	0.051	0.949		
3	14,445.30	14,547.38	14,477.52	0.80	342.30 (0.001)	176 (23.0%)	0.895	0.105	0.000	
						386 (50.5%)	0.068	0.901	0.031	
						203 (26.5%)	0.000	0.077	0.923	
4	14,358.69	14,488.60	14,399.69	0.77	96.20 (0.072)	101 (13.2%)	0.882	0.000	0.000	0.118
						99 (12.9%)	0.000	0.895	0.105	0.000
						208 (27.2%)	0.000	0.069	0.834	0.097
						357 (46.7%)	0.057	0.000	0.068	0.088

Note. AIC = Akaike Information Criterion; BIC = Bayesian Information Criterion; SABIC = Sample-Adjusted Bayesian Information Criterion; LMRT = Lo-Mendell-Rubin adjusted likelihood ratio test.

**Table 3:** Head-to-head comparison of predictive utility of extracted personality profiles versus Big Five personality traits.

Outcomes	Model 1		Model 2	
	Profiles	Traits	Traits	Profiles
**Depression**	0.015	0.015 *	0.028	0.003
**Anxiety**	0.028	0.011	0.030	0.009 *
**PTSD symptoms**	0.034	0.014	0.043	0.004
**OCD symptoms**	0.006	0.014	0.017	0.004

* *p* < 0.05.

**Table 4:** Profiles’ mean and standard deviations of the Big Five personality traits.

Profiles	*n*	Extraversion	Agreeableness	Conscientiousness	Neuroticism	Openness
Maladaptive	176	4.60 (1.33)	3.89 (1.07)	4.57 (1.33)	7.81 (1.14)	4.69 (1.34)
Adaptive	386	6.52 (1.47)	6.11 (1.42)	6.19 (1.43)	5.87 (1.36)	6.23 (1.41)
Highly adaptive	203	8.42 (1.24)	8.45 (1.24)	8.48 (1.17)	3.41 (1.13)	8.48 (1.21)

**Table 5:** Mean differences among latent personality profiles in anxiety, depression, PTSD, and OCD symptoms, and mental well-being.

	Maladaptive	Adaptive	Highly Adaptive	F-Value (sig.)	Partial Eta Squared
	M (SD)	M (SD)	M (SD)		
**Anxiety**	7.46(6.25)	7.53 (5.46)	5.41 (4.79)	10.15 (<0.001)	0.028
**Depression**	9.59 (7.19)	9.36 (6.09)	7.66 (5.89)	5.04 (0.003)	0.015
**PTSD symptoms**	42.04 (18.09)	41.23 (14.51)	35.36 (11.13)	13.22 (<0.001)	0.034
**OCD symptoms**	10.98 (7.49)	11.71 (6.69)	10.44 (6.44)	2.43 (0.088)	0.006

Note. *M* = mean; *SD* = standard deviation; Pillai’s F (12, 1516) = 7.078, *p* < 0.001; eta squared = 0.053; Observed power = 1.00.

**Table 6:** Post hoc test among latent profiles in anxiety, depression, and PTSD symptoms, and mental well-being.

Dependent Variable	(I) Latent Profiles	(J) Latent Profiles	Mean Difference (I–J)	Sig.	95% Confidence Interval	
					Lower Bound	Upper Bound
**Anxiety**	Maladaptive	Adaptive	−0.07	0.887	−1.05	0.91
		Highly adaptive	2.05	<0.001	0.94	3.16
	Adaptive	Highly adaptive	2.12	<0.001	1.19	3.06
**Depression**	Maladaptive	Adaptive	0.23	0.692	−0.90	1.35
		Highly adaptive	1.93	0.003	0.65	3.20
	Adaptive	Highly adaptive	1.70	0.002	0.62	2.77
**PTSD symptoms**	Maladaptive	Adaptive	0.81	0.541	−1.80	3.43
		Highly adaptive	6.68	<0.001	3.72	9.64
	Adaptive	Highly adaptive	5.87	<0.001	3.37	8.36

## Data Availability

The data presented in this study are available on request from the corresponding author.
